# Roles of SPI-2 T3SS effectors in virulence of *Salmonella* Choleraesuis and Construction of a triple-gene mutant vaccine strain

**DOI:** 10.3389/fvets.2025.1637327

**Published:** 2025-08-12

**Authors:** Rui Xu, Xiangfei Ji, Junqi Lian, Dekang Zhu, Mafeng Liu, Mingshu Wang, Renyong Jia, Shun Chen, Qiao Yang, Ying Wu, Shaqiu Zhang, Juan Huang, Xumin Ou, Di Sun, Bin Tian, Yu He, Zhen Wu, Anchun Cheng, Xinxin Zhao

**Affiliations:** 1 Institute of Veterinary Medicine and Immunology, College of Veterinary Medicine, Sichuan Agricultural University, Chengdu, Sichuan, China; 2 Research Center of Avian Diseases, College of Veterinary Medicine, Sichuan Agricultural University, Chengdu, Sichuan, China; 3 Key Laboratory of Animal Disease and Human Health of Sichuan Province, Chengdu, Sichuan, China; 4 Engineering Research Center of Southwest Animal Disease Prevention and Control Technology, Ministry of Education of the People's Republic of China, Chengdu, Sichuan, China; 5 Hulunbuir Agricultural and Livestock Product Quality and Safety Center, Hulunbuir, Inner Mongolia, China

**Keywords:** *S. choleraesuis*, SPI-2, type III secretion system, virulence, vaccines

## Abstract

Effector protein functions of Type III secretion system (T3SS) encoded by *Salmonella* pathogenicity islands 2 (SPI-2) have not been fully characterized in *Salmonella enterica* serovar Choleraesuis. This study characterized 21 effectors of SPI-2 T3SS of *S. Choleraesuis* in terms of macrophage survival and virulence in mice via construction of various gene mutant strains. Eight effector genes including *sseF, sseJ, sifB, sseK, sifA, sopD*_2_, *steC*, and *steD* contributed to bacterial survival in macrophage cell line RAW264.7; whereas only *sopD*_2_ also promoted bacterial virulence in mice like other three effector genes *sseL, steA*, and *spiC*. The mutant strain, Δ*sopD*_2_, Δ*sseL*, Δ*steA*, or Δ*spiC*, led to higher mouse survival compared to the wild-type strain post-oral infection, while their bacterial loads in spleen and liver were not reduced except the Δ*spiC* that was undetectable in mouse tissues. Then, the triple-gene mutant strain Δ*sseL*Δ*sopD*_2_Δ*steA* was constructed and found to be virulence attenuated with a compromised colonization ability. Finally, immunization of this mutant orally induced robust serum IgG responses and provided 40% protection against lethal *S. Choleraesuis* challenge. Our study highlights the critical role of four SPI-2 T3SS effectors in *S. Choleraesuis* pathogenesis.

## 1 Introduction

*Salmonella enterica* serovar Choleraesuis (*S. Choleraesuis*) is a zoonotic pathogen causing swine paratyphoid, characterized by enterocolitis and septicemia, which imposes substantial economic burdens on global swine husbandry (1, 2). Although *S. Choleraesuis* is adapted to pigs, it is also a major cause of life-threatening septicemia, particularly in children and immunocompromised individuals in East Asia and Europe (3, 4). Human infections frequently arise from direct contact with infected swine or ingestion of contaminated pork-derived products (1, 5). Due to the excessive use of antibiotics and environmental diversity, the emergence of multidrug-resistant *S. Choleraesuis* strains has become increasingly prevalent (6–8).

Vaccination represents the most cost-effective prophylactic strategy for disease control, effectively reducing antibiotic use and retarding the emergence of antibiotic resistance (9). The live vaccine strain C500 obtained by chemical mutagenesis has been used in China for more than 40 years to prevent paratyphoid fever in piglets (10). However, it still has non-negligible side effects related to residual toxicity, leading to adverse reactions in animals after vaccination and the genetic background of the vaccine is still unclear (11). Notably, there is no available vaccine for human use to date. Therefore, it is urgent and necessary to devise an innovative and efficacious vaccine against this important pathogen.

Understanding the mechanisms underlying bacterial pathogenesis is essential for the development of live attenuated vaccines. The *Salmonella* pathogenicity islands 2 (SPI-2) type III secretion system (T3SS) has been found to be essential for bacterial virulence of *S*. Typhimurium (12). This system promotes bacterial replication within membrane-bound *Salmonella*-containing vesicles (SCV) in host macrophages via production of various effector proteins (13). Loss of some effector genes *sseF, sseG*, and *sseM* that maintain bacterial nutrient acquisition within vesicles significantly reduces the replication ability of *S*. Typhimurium in host cells (14, 15). Effectors SpvB and SteC manipulate the actin cytoskeleton, affecting bacterial replication and subsequently impacting bacterial virulence (16, 17). Mutations in SPI-2 T3SS effectors can attenuate virulence, positioning them as promising candidates for live attenuated vaccines (18).

Despite their recognized importance, the specific roles of individual SPI-2 T3SS effectors in *S. Choleraesuis* virulence remain poorly characterized. This study aims to characterize SPI-2 T3SS effectors of *S. Choleraesuis* via construction of various single effector gene deletion strains. The mutant strains were systematically evaluated for their intracellular survival in macrophages, growth curves and swimming, and virulence in mice. Then, a triple mutant strain (Δ*sseL*Δ*sopD*_2_Δ*steA*) was constructed based on virulence effectors and its protection efficacy was finally evaluated.

## 2 Materials and methods

### 2.1 Bacterial strains and growth conditions

A complete list of all bacterial strains and plasmids utilized in the experiments is provided in Supplementary Tables S1, S2. *Salmonella* Choleraesuis CVCC2139 was referred to as the wild-type (WT) strain for genetic manipulation to construct the indicated mutants. The *Escherichia coli* (*E. coli*) SM10 λ pir strain (19) served as the host for transferring suicide plasmids. All bacterial strains were cultured in Luria Bertani (LB) broth or agar at 37°C containing the appropriate antibiotics: 25 μg/mL chloramphenicol, 100 μg/mL ampicillin, and 50 μg/mL 2,6-diaminopimelic acid. For counterselecting mutant constructs via the *sacB* gene system, NaCl-free LB agar supplemented with 12.5% (w/v) sucrose was used.

### 2.2 Construction of the *S. Choleraesuis* mutant and complemented strains

Twenty-two *S. Choleraesuis* mutants were generated via allelic exchange, employing the suicide plasmid pRE112 as previously detailed (20). The primer sequences designed for gene deletion or complementation in *S. Choleraesuis* strains are detailed in Supplementary Table S3. To generate the Δ*sseJ* mutant, upstream and downstream homologous arms were PCR-amplified using primer pairs D*sseJ*-1F/1R and D*sseJ*-2F/2R, respectively. These PCR products were joined by overlap PCR and subsequently cloned and inserted into the suicide vector pRE112 (21) through seamless cloning generating plasmid pRE112-Δ*sseJ*, which carries a deletion of the entire *sseJ* gene. The pRE112-Δ*sseJ* plasmid was subsequently introduced into the WT strain via conjugation. This process involved chloramphenicol-mediated positive selection and *sacB*-mediated sucrose sensitivity screening for the generation of the markerless mutant strain Δ*sseJ*. Furthermore, to complement *sseJ* gene in the Δ*sseJ*, the coding sequence of *sseJ* were amplified with the primer *sseJ-*F/R. Then, the PCR product was inserted into the plasmid of pCZb1 (20) via a Seamless Cloning Kit (Sangon Biotech, Shanghai, China), generating plasmid pCZb1-*sseJ*. Following, the recombinant plasmid was transformed into the mutant strain Δ*sseJ* to construct the complemented strain named C-Δ*sseJ*. The same method was applied to constructions of other gene mutants and complemented strains.

### 2.3 Detection of growth curves of *S. Choleraesuis* strains

The *S. Choleraesuis* WT and mutant strains were inoculated in 5 mL of LB broth at 37°C with shaking at 180 rpm/min overnight. The following day, cultures of each strain were normalized to an OD_600_ of 0.05 and then cultured in LB broth at 37°C with shaking at 180 rpm/min. The OD_600_ of each culture was measured every 2 h for 12 h.

### 2.4 Swimming assay

The swimming motility phenotypes of wild-type (WT) and mutant bacterial strains were evaluated using a previously described protocol (22). Cultures of each strain were grown in LB broth to an optical density (OD_600_) of 0.6–0.8. Bacteria were harvested by centrifugation, washed twice with PBS, and resuspended in the same buffer. Subsequently, 3 μL of the bacterial suspension was applied as droplets to LB agar plates containing 0.25% agar. After incubation at 37°C for 6 h, the diameter of the bacterial migration zone was measured to assess swimming motility.

### 2.5 Adhesion, invasion and intracellular survival of *S. Choleraesuis* in macrophages

RAW264.7 macrophages were plated at a density of 5 × 10^5^ cells per well in 24-well plates containing DMEM (Gibco, NY, USA) supplemented with 10% FBS (Tian Hang, Hangzhou, China) and 1% penicillin-streptomycin. *S. Choleraesuis* WT or mutant strains were added at a multiplicity of infection (MOI) of 100. Following a 30-min incubation in a 5% CO_2_ at 37°C incubator to facilitate bacterial adhesion, cell monolayers were washed thrice with PBS to remove non-adherent bacterial cells. The adherent bacteria were then released by lysing the cells with 0.2% Triton X-100, and their numbers were enumerated via serial dilution and colony counting.

For the invasion assay, after the 30-min adhesion step, fresh DMEM was added and cells were incubated for an additional 90 min at 37°C under 5% CO_2_. After incubation, the supernatant was discarded, cells were washed twice with PBS and lysed using 0.2% Triton X-100 to enumerate intracellular bacteria.

For intracellular survival analysis, following the invasion step, DMEM supplemented with 10 ng/mL gentamicin was used to eliminate extracellular bacteria. *T* = 0 h was defined as the initial time point following invasion. At designated time points (*T* = 0 h and 24 h), serial dilutions of the resulting cell lysates were then plated onto MacConkey agar (Coolaber, Beijing, China) plates for bacterial enumeration and incubated at 37°C for 24 h to count colony-forming units (CFUs).

### 2.6 Colonization and virulence of *S. Choleraesuis* mutant strains in mice

Female Kunming mice (6 weeks old) were procured from Dashuo Experimental Animal Ltd. (Chengdu, China) and underwent a 1-week acclimation period before experimental procedures. Bacterial colonization and virulence phenotypes were evaluated using methodologies reported in prior studies (20, 22). Ten mice were orally inoculated with PBS or approximately 1 × 10^8^ CFU of *S. Choleraesuis* WT strain or each mutant strain. Then, spleens and livers were collected from 5 mice at 6 days post-infection. The samples were weighed, ground in PBS, and the bacterial suspensions were serially diluted and spread onto MacConkey agar (Coolaber, Beijing, China) to enumerate viable CFUs, which were expressed as log_10_ CFU/g. The remaining 5 mice in each group were observed for survival for 1 month after infection.

### 2.7 Measurement of 50% Lethal dose (LD_50_) of the Δ*sseL*Δ*sopD_2_*Δ*steA* strain

The LD_50_ of the Δ*sseL*Δ*sopD*_2_Δ*steA* was determined as previously described (23). 10-fold serial dilutions of the CFU of the Δ*sseL*Δ*sopD*_2_Δ*steA* strain were orally inoculated into groups of Kunming mice (*n* = 5/per dose). Animals were monitored daily for 30 days post-infection to assess survival rates. The median lethal dose (LD_50_) was determined using the Reed-Muench method. To ensure humane endpoints, mice exhibiting severe distress—characterized by labored breathing, tremors, unresponsiveness to tactile stimuli, or inability to access food/water—were humanely euthanized via CO_2_ inhalation. Deceased animals were immediately subjected to sterilization, sealed in biohazard bags, and transferred to the Sichuan Agricultural University Laboratory Animal Center for compliant biosafety disposal.

### 2.8 Immunization and challenge

Female Kunming mice (6–8 weeks old) were randomly divided into three groups (*n* = 20/group). The experimental group received an oral gavage of 1 × 10^9^ CFU Δ*sseL*Δ*sopD*_2_Δ*steA* in 200 μL PBS, while the control group received an equal volume of PBS alone. A booster immunization was administered on day 14 using the same protocol. Serum samples were collected via retro-orbital bleeding on days 7 and 21 from six randomly selected mice per group. On day 42, all mice were orally challenged with 10-fold LD_50_ of *S. Choleraesuis* CVCC2139. Five mice per group were euthanized on day 6 post-challenge, and samples from the spleen and liver were collected for measurement of bacterial loads. Survival of the remaining mice was monitored and recorded daily for 30 days.

### 2.9 Enzyme-linked immunosorbent assay (ELISA)

Antibody titers against inactivated *S. Choleraesuis* antigens were quantified using an indirect ELISA protocol as previously described (24). In brief, 100 μL of 10^9^ CFU/ml of the heat-killed *S. Choleraesuis* antigens was added to wells of a 96-well ELISA plate coated with antigen with overnight incubation at 4°C. The next day, the plates were washed three times with PBST followed by blocking with 5% BSA (BD, San Diego, CA) in PBS at 37°C for 2 h. Following antigen coating and blocking, the plate was washed three times with PBST. Serum samples, diluted 1:200 in blocking buffer (5% BSA in PBS), were added to each well (100 μL/well) and incubated at 37°C for 1 h in a humidified chamber. The plate was then washed five times with PBST to remove unbound antibodies. Subsequently, 100 μL of HRP-conjugated goat anti-mouse IgG (Abclonal, Wuhan, China), diluted 1:5,000 in antibody diluent, was added to each well and incubated at 37°C for 1 h. After five additional PBST washes, the plate was ready for substrate development. 100 μL of TMB substrate solution (Macgene, Shanghai, China) was added, and the plates were incubated in the dark at 25°C for 10 min. After adding 50 μL of 2 M H_2_SO_4_ to stop the reaction, absorbance was measured at 450 nm using a Bio-Rad microplate reader (Bio-Rad, California, USA).

### 2.10 Ethics statement

All animal procedures were conducted in strict adherence to the Guide for the Care and Use of Laboratory Animals published by China's Ministry of Science and Technology. The study protocol was approved by the Animal Ethics Committee of Sichuan Agricultural University and the Sichuan Laboratory Animal Management Committee (permit number: SYXK2019-187), ensuring compliance with national and institutional welfare guidelines.

### 2.11 Statistical analysis

Data are presented as the mean ± standard deviation (SD) and analyzed using one-way analysis of variance (ANOVA) followed by Tukey's *post-hoc* multiple-comparison test with GraphPad Prism software (GraphPad Software, California, USA). Statistical significance was defined as *P* < 0.05. All *in vitro* experiments were independently repeated three times to ensure reproducibility.

## 3 Results

### 3.1 Roles of SPI-2 T3SS effectors of *S. Choleraesuis* in bacterial adhesion to, invasion into and survival within macrophages

**Figure 1:**
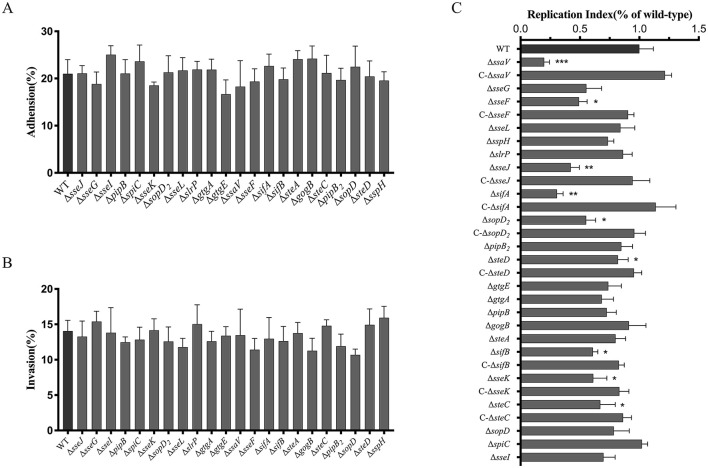
Roles of SPI-2 T3SS effectors of *S. Choleraesuis* in bacterial adhesion to **(A)**, invasion into **(B)** and survival **(C)** within macrophages. **(A and B)** RAW264.7 cells were infected with WT or mutant strains (MOI = 100) for 30 min at 37°C. After PBS washing, adherent bacteria were quantified by direct lysis and CFU counting. The adhesion rate = (*N*_adherent_/*N*_initial_ × 100%). For invasion, cells were further incubated for 1 h to invade, then lysed by adding 0.2% triton X-100 then lysed for CFU counts. The invasion rate = (*N*_invasive_/*N*_adherent_ × 100%). C. For intracellular survival, invaded cells by the *S. Choleraesuis* were further cultured for 24 h. Then cells were lysed and bacterial counts were measured. Intracellular bacteria were quantified as replication index (*N*_survival_/*N*_adherent_ × 100%). Asterisks above the error bars indicate significant differences compared with the WT group. *P < 0.05; **P < 0.01; ***P < 0.001.

Twenty-one effector genes (*sseJ, sseG, slrP, sseF, gtgE, gogB, sspH, gtgA, sifA, sifB, sseK, steA, steC, sseL, sopD*_2_, *spiC, sseI, pipB, pipB*_2_, *sopD, steD*) were screened and each of them was deleted from the WT *S. Choleraesuis* strain CVCC2139. The *ssaV* mutant strain (Δ*ssaV*) was also constructed as a positive control as the SsaV is a structural component forming the inner ring of the SPI-2 T3SS injectosome that is involved in effector protein translocation (25). Then, the mutant strains were compared to the WT strain in terms of the ability of bacteria to adhere to, invade and survive within RAW264.7 macrophages. Deletion of the SPI-2 T3SS effector genes neither affected bacterial adhesion to nor changed bacterial invasion into macrophages (Figures 1A, B). Nevertheless, bacterial replication in the mutant strain Δ*ssaV*, Δ*sseF*, Δ*sseJ*, Δ*sifB*, Δ*sseK*, Δ*sifA*, Δ*sopD*_2_, Δ*steC*, or Δ*steD* was significantly decreased compared to that in the WT strain, while loss of either of the other 13 genes had on adverse effects (Figure 1C). Gene complementation *in trans* in the mutant strains fully or partially restored the WT phenotypes (Figure 1C). This finding indicated that effector genes including *sseF, sseJ, sifB, sseK, sifA, sopD*_2_, *steC*, and *steD* promoted bacterial survival in macrophages.

### 3.2 Roles of SPI-2 T3SS effectors of *S. Choleraesuis* in bacterial growth and swimming

**Figure 2:**
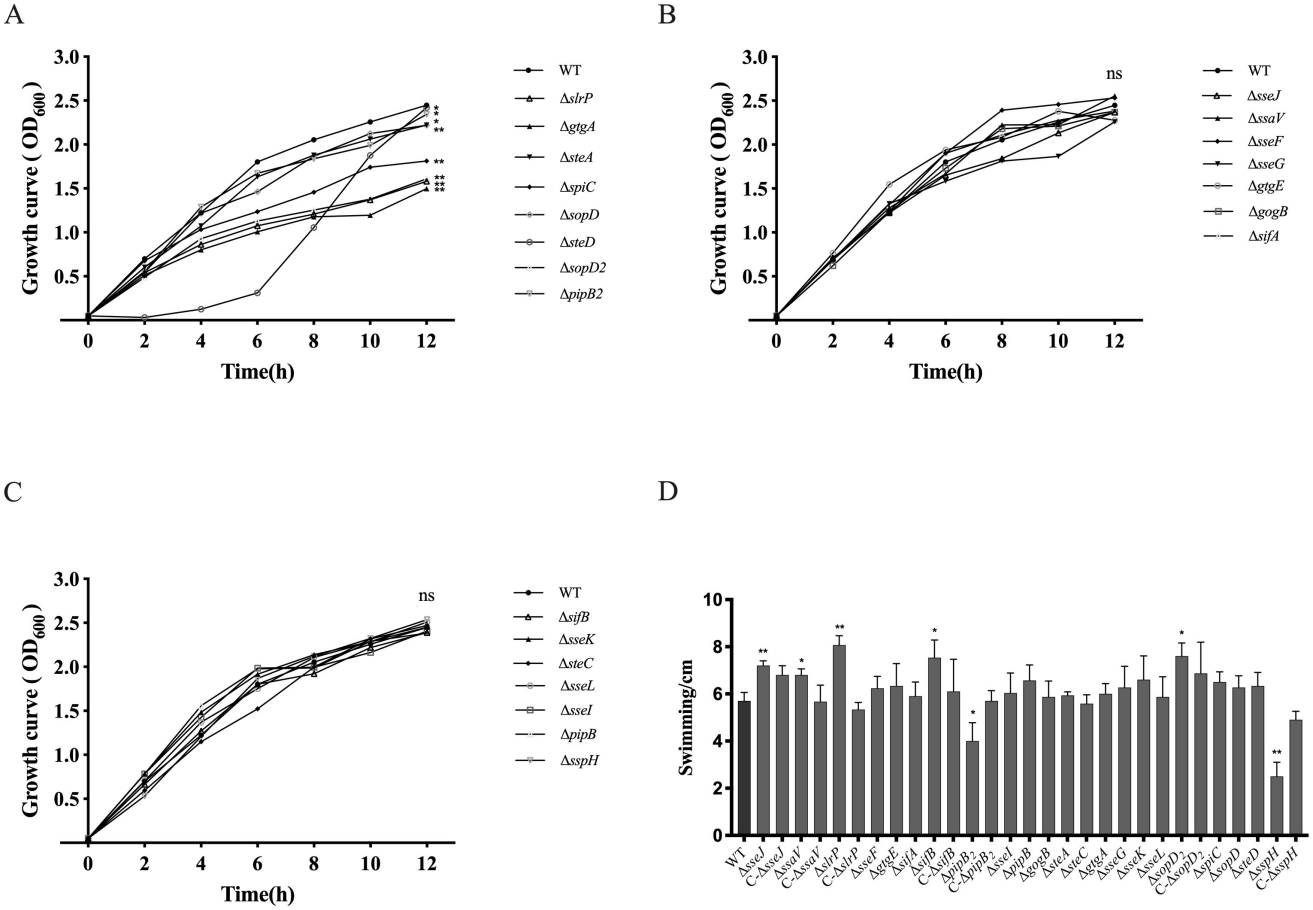
Detection of growth curves and swimming of *S. Choleraesuis* strains. **(A–C)** Growth curves of *S. Choleraesuis* WT and mutant strains were analyzed by measuring OD_600_ every 2 h for 12 h. **(D)** Swimming assay. The WT strain, 22 mutant strains and complemented strains were cultured in LB to OD_600_ = 0.6–0.8, harvested and suspended in PBS. Three microliters of bacterial suspension were spotted onto the center of a soft agar plate. Then, colony diameters were measured after 6 h of incubation at 37°C. An asterisk above the error bar shows a significant difference from the WT group. *P < 0.05; **P < 0.01.

The 21 *S. Choleraesuis* mutant strains were subjected to detection of growth curves and swimming. Compared to the WT strain, the growth rates of the Δ*gtgA*, Δ*steA*, Δ*spiC*, Δ*sopD*_2_, Δ*slrP*, Δ*pipB*_2_, Δ*sopD*, and Δ*steD* strains were significantly reduced in LB broth at 37°C (Figure 2A). In contrast, the remaining 14 mutants exhibited similar growth rates to the WT strain (Figures 2B, C). This finding suggested that the effector genes *gtgA, steA, spiC, sopD*_2_, *slrP, pipB*_2_, *sopD*, and *steD* promotes the *in vitro* growth of *S. Choleraesuis*. Also, swimming of the Δ*sseJ*, Δ*slrP*, Δ*sifB*, and Δ*sopD*_2_ mutants were significantly enhanced compared to that of the WT strain, whereas the Δ*pipB*_2_ and Δ*sspH* strains exhibited reduced motility (Figure 2D). Complementation of *sseJ, slrP, sifB, sopD*_2_, *pipB*_2_and *sspH* in corresponding mutants restored WT swimming phenotype. Therefore, the effector genes *sseJ, slrP, sifB*, and *sopD*_2_ restrain the swimming ability of *S. Choleraesuis*, whereas *sspH* and *pipB*_2_ positively influence motility.

### 3.3 Roles of SPI-2 T3SS effectors of *S. Choleraesuis* in bacterial virulence in mice

**Figure 3:**
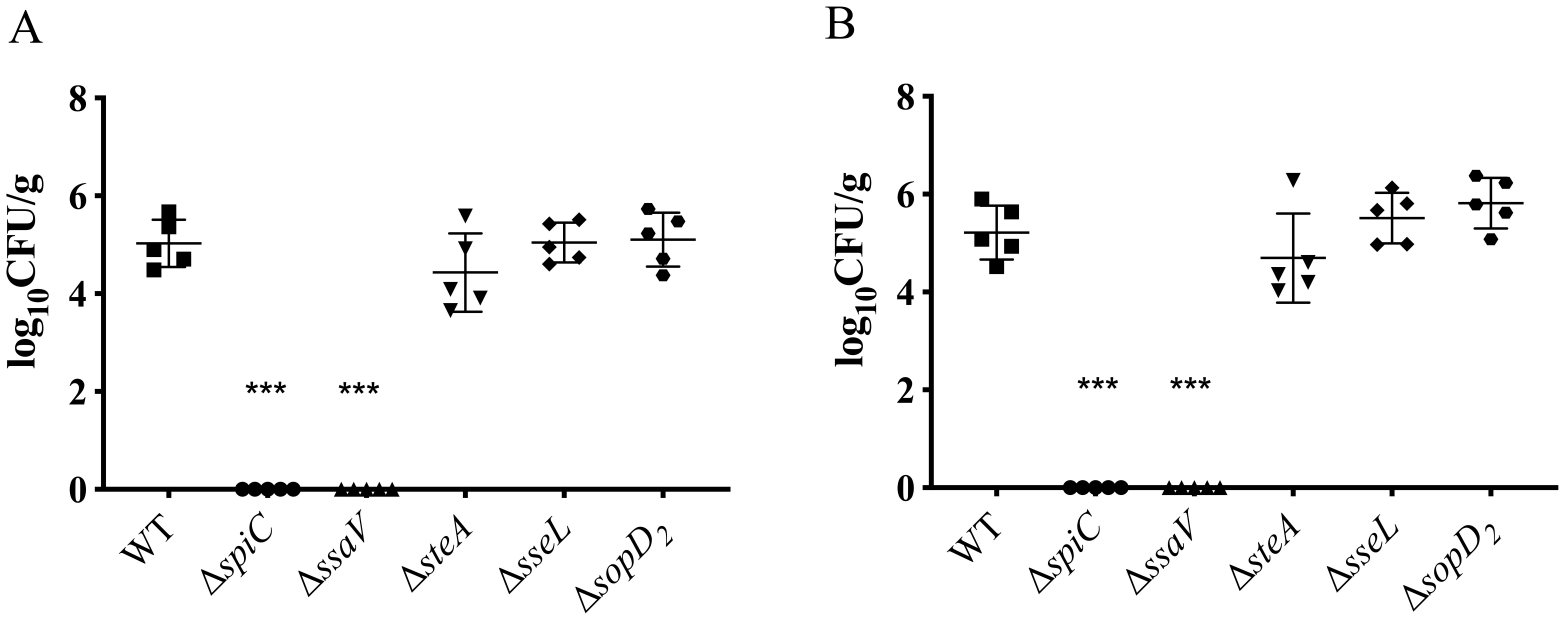
Detection of bacterial loads in mice after infection with *S. Choleraesuis* strains. Kunming mice (*n* = 5/group) were inoculated with *S. Choleraesuis* WT strain and mutant strains orally, respectively. Then, the bacterial loads in spleens **(A)** and livers **(B)** were measured and calculated as log_10_ CFU/g. An **asterisk** above the error bar indicates a significant difference from the WT group. ***P < 0.001.

To determine roles of the SPI-2 T3SS effector in virulence of *S. Choleraesuis*, mice were orally administered with 1 × 10^8^ CFU of the WT strain or each of the mutant strains. The survival of mice was monitored for 1 month. The WT strain led to 40% of survival post-infection, while the five strains including Δ*spiC*, Δ*ssaV*, Δ*sseL*, Δ*sopD*_2_, and Δ*steA* caused no death (Table 1). Also, Δ*gtgA*, Δ*gtgE*, Δ*sseF*, Δ*sifA*, and Δ*sspH* resulted in increased survival (80%), by contrast, all mice succumbed to the Δ*steD* infection. The remaining strains caused a 40% or 60% survival (Table 1). Thus, SPI-2 T3SS effector genes *sseL, sopD*_2_, and *spiC*, and the gene *ssaV* contributed remarkably to *S. Choleraesuis* virulence in mice, functioning as virulence genes. To further detect the roles of the five virulence genes in bacterial colonization, mice were inoculated with 10^8^ CFU of the WT or mutant strains, then bacterial loads in liver and spleen were measured 6 days post-infection. No bacteria were recovered from the Δ*spiC* and Δ*ssaV* groups. However, the bacterial loads of Δ*sseL*, Δs*opD*_2_, and Δ*steA* in liver and spleen tissues were comparable to those of the wild-type strain (Figures 3A, B). This finding suggests that the *spiC* and *ssaV* contribute to the colonization of *S. Choleraesuis* in the liver and spleen of mice, while *sseL, sopD*_2_, and *steA* are not involved in bacterial colonization.

**Table 1:** Survival rates of mice infected with *S. Choleraesuis* strains.

Strains	Challenge dose (CFU)	Survival	Survival rate
WT	10^8^	2/5	40%
Δ*spiC*		5/5	100%
Δ*sseL*		5/5	100%
Δ*sopD_2_*		5/5	100%
Δ*steA*		5/5	100%
Δ*ssaV*		5/5	100%
Δ*slrP*		3/5	60%
Δ*gogB*		3/5	60%
Δ*sseJ*		2/5	40%
Δ*sopD*		3/5	60%
Δ*sseI*		2/5	40%
Δ*gtgA*		4/5	80%
Δ*gtgE*		4/5	80%
Δ*sseG*		2/5	40%
Δ*sseF*		4/5	80%
Δ*sifA*		4/5	80%
Δ*sifB*		3/5	60%
Δ*sseK*		3/5	60%
Δ*pipB*		2/5	40%
Δ*steC*		3/5	60%
Δ*pipB_2_*		2/5	40%
Δ*steD*		0/5	0%
Δ*sspH*		4/5	80%
PBS		5/5	100%

### 3.4 Virulence and colonization of the triple mutant strain ΔsseLΔsopD_2_ΔsteA of *S. Choleraesuis* mutants in mice

**Figure 4:**
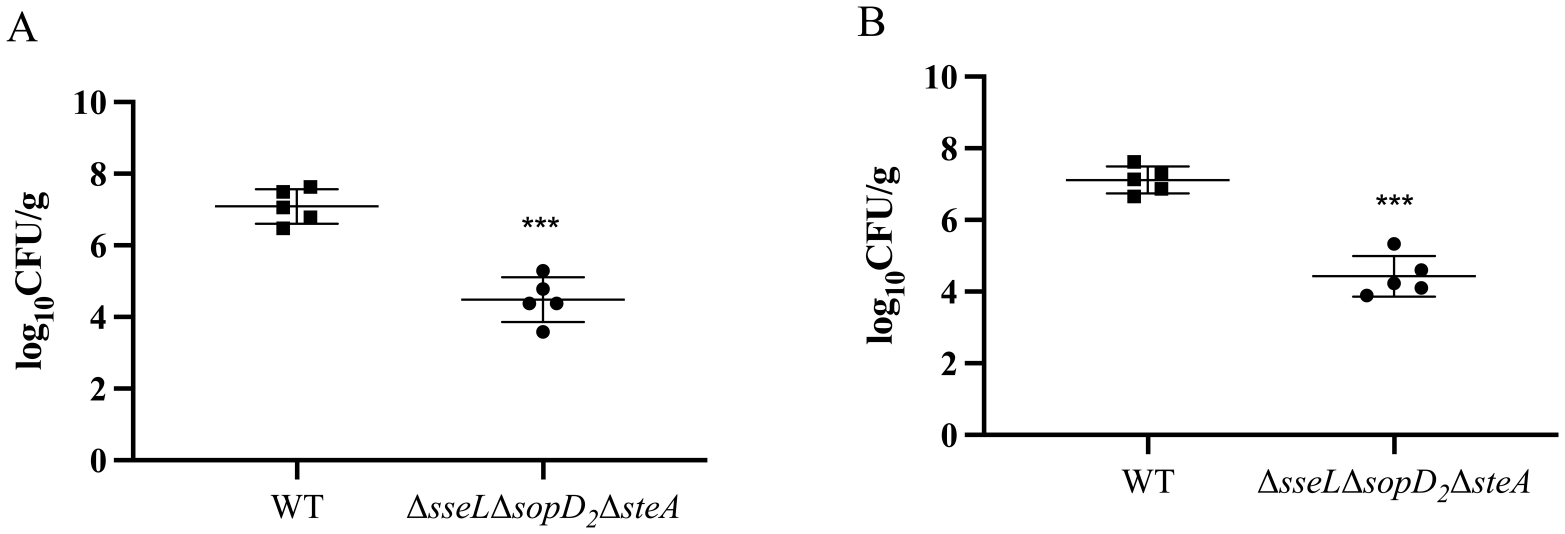
Colonization of the triple mutant strain Δ*sseL*Δ*sopD*_2_Δ*steA* of *S. Choleraesuis* in mice. Mice were inoculated with *S. Choleraesuis* WT strain or the Δ*sseL*Δ*sopD*_2_Δ*steA* orally. Bacterial loads in spleen **(A)** and liver **(B)** tissues were quantified as log_10_ CFU/g. The **asterisk** above the error bar indicates a significant difference compared with the WT group. ***P < 0.001.

Although the two genes *spiC* and *ssaV* play a significant role in bacterial virulence, their mutant strains lost colonization ability in mice, hinting their poor immunogenicity. To develop a suitable live attenuated strain, the other three virulence genes *sseL*, s*opD*_2_, and *steA* were deleted from the WT strain simultaneously, generating a triple mutant Δ*sseL*Δ*sopD*_2_Δ*steA*. This strain colonized of the spleen and liver at a significantly lower level than the WT strain post oral infection (Figure 4). Also, the LD_50_ of the triple mutant was >1.65 × 10^10^ CFU, demonstrating at least a 150-fold reduction in virulence compared with that of the wild-type strain with the LD_50_ of 1.08 × 10^8^ CFU (Table 2).

**Table 2:** The LD_50_ of the *S. Choleraesuis* strains.

Strains	Challenge dose (CFU) and death					LD_50_(CFU)
	10^6^	10^7^	10^8^	10^9^	10^10^	
WT	0/5	2/5	3/5	5/5	5/5	1.08 × 10^8^
Δ*sseL*Δ*sopD_2_*Δ*steA*	0/5	0/5	0/5	0/5	1/5	>1.65 × 10^10^
PBS					0/5	

### 3.5 Protection efficacy of the triple mutant strain ΔsseLΔsopD_2_ΔsteA

**Figure 5:**
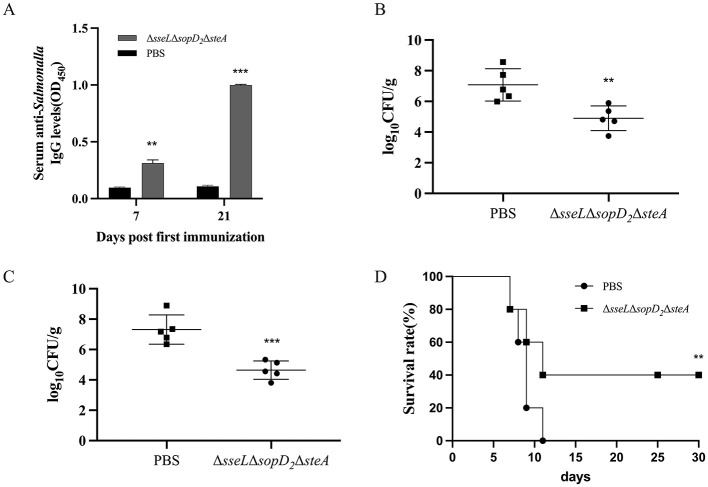
Antibody responses and protection efficacy induced by the triple mutant strain Δ*sseL*Δ*sopD*_2_Δ*steA*. Mice were immunized orally with the attenuated strain Δ*sseL*Δ*sopD*_2_Δ*steA* twice and then challenged with the *S. Choleraesuis* WT strain 28 days post-second immunization. Indirect ELISA was used to detect serum IgG levels against whole bacterial antigens 7 days and 21 days post-immunization **(A)**. Bacterial loads in the spleen **(B)** and liver **(C)** were measured 6 days post-challenge. **(D)** Survival rates of mice were monitored over a 30-day period after challenge. **Asterisks** above the error bars indicate significant differences compared with the PBS group. **P < 0.01; ***P < 0.001.

To evaluate the vaccine potential of the attenuated strain Δ*sseL*Δ*sopD*_2_Δ*steA*, mice were orally administered with 10^9^ CFU of the vaccine strain twice with an interval of 14 days and then were challenged orally with a lethal dose of the *S. Choleraesuis* WT strain 28 days post-second immunization. Immunization with the Δ*sseL*Δ*sopD*_2_Δ*steA* induced significantly higher serum IgG responses to whole bacterial antigens than with the PBS group on Day 7 and 21 post-immunization (Figure 5A). Following the challenge, the bacterial loads in the spleen and liver of the Δ*sseL*Δ*sopD*_2_Δ*steA* group were significantly lower than those in the PBS group (Figures 5B, C). Furthermore, all the mice in the PBS control group died, whereas 40% of the mice in the immunized group survived (Figure 5D). Thus, immunization with the Δ*sseL*Δ*sopD*_2_Δ*steA* strain induced a robust antibody response, significantly reduced the tissue loads of the challenge strain, and provided 40% protection efficacy against lethal *S. Choleraesuis* infection.

## 4 Discussion

Our comprehensive analysis of SPI-2 T3SS effectors in *S. Choleraesuis* reveals multifaceted roles of individual effectors in intracellular survival, systemic virulence, motility, and growth, offering mechanistic insights into how this pathogen adapts to host defenses and establishes infection.

None of detected SPI-2 T3SS effectors was involved in the adhesion and invasion process of *S. Choleraesuis* to the macrophage RAW264.7, which is in line with previous studies on *S*. Typhimurium (26–29). We also observed notable effects of effectors on bacterial motility and growth. Several mutants exhibited reduced growth rates or altered swimming motility, indicating that SPI-2 effectors also influence global physiological fitness. Such effects may be mediated via metabolic reprogramming or indirect transcriptional regulation. For instance, *pipB*_2_ has been shown to alter host cytoskeletal tension and organelle dynamics, which may feed back to bacterial stress signaling (30). Reduced motility may compromise their ability to penetrate mucus layers or disseminate systemically, further contributing to attenuation (31). The *spiC* mutant strain of *S. Enteritidis* exhibits stronger swimming ability (32); whereas deletion of the *spiC* of *S. Choleraesuis* did not affect swimming. These contradictions suggested that some effector molecules exhibit functional heterogeneity across different *Salmonella* serovars.

Eight effector genes *sseF, sseJ, sifB, sseK, sifA, sopD*_2_, *steC*, and *steD* significantly enhanced the survival of *S. Choleraesuis* in macrophages, which highlights the core function of SPI-2 T3SS effector in maintaining intracellular survival and is largely consistent with previous studies. Most of these effectors are known to modulate SCV maturation, membrane dynamics, or host trafficking pathways, helping bacteria to evade lysosomal degradation and acquire nutrients. For instance, *sifA* stabilizes the SCV membrane and recruits kinesin-1 via SKIP (33), while *sseF* and *sseG* facilitate microenvironment construction (34). The involvement of *sopD*_2_ and steD suggests a multi-effector strategy to subvert host immunity: *sopD*_2_ interferes with Rab GTPase-mediated trafficking (35), while *steD* downregulates MHC II surface expression through host ubiquitination machinery (36). However, SteC of *S*. Typhimurium barely affects bacterial proliferation in macrophages (37). Interestingly, *steA* and *pipB*_2_, although previously implicated in vacuole positioning and actin remodeling (30, 38), had minimal impact on intracellular survival in *S. Choleraesuis*, hinting at possible functional redundancy or compensation by other effectors in this serovar.

All the effectors associated with intracellular survival except for *sseJ* and *steD* contribute to the virulence of *S. Choleraesuis* in mice, while three virulence determinants (*spiC, sseL*, and *steA*) were not related to bacterial intracellular survival. A previous study also found that protein SseL was shown to enhance the virulence of *S. Pullorum* by suppressing host NF-κB signaling but not affect the intracellular bacterial survival (39). These findings indicated a lack of strong correlation between intracellular replicative capacity and systemic virulence.

Construction of live attenuated bacterial vaccines should balance the virulence and immunogenicity (40). However, we found that the Δ*spiC* of *S. Choleraesuis* as well as the Δ*ssaV* was avirulent and colonized mouse liver or spleen at levels below the threshold of detection. Too-low bacterial loads in tissues imply low immunogenicity (41). Therefore, the two strains were not considered as vaccine candidates in our study. Compared to the currently licensed live attenuated vaccine strain C500 in China, which was derived through chemical mutagenesis and has been used for over four decades, the Δ*sseL*Δ*sopD*_2_Δ*steA* strain developed in this study presents both advantages and limitations. C500 has demonstrated high protection efficacy in piglets but suffers from residual virulence and has an undefined genetic background, which raise biosafety concerns and hinder its broader application, particularly in immunocompromised hosts (10). In contrast, the triple mutant strain constructed here is genetically defined and rationally attenuated by deletion of three characterized virulence genes, thereby reducing the risk of reversion and enhancing safety. However, immunization with Δ*sseL*Δ*sopD*_2_Δ*steA* conferred only moderate protection (40%) against lethal challenge in mice, which is lower than the reported protection level of C500 or other strains such as the Δ*spiC* mutant of *S. Pullorum* that offered more than 90% protection (18). This limited efficacy may be attributed to its moderate tissue colonization and immunogenicity. Therefore, further optimization is required, such as incorporating additional immunostimulatory mutations or adjuvant delivery strategies, to enhance both antigen persistence and immune protection.

## Data Availability

The original contributions presented in the study are included in the article/Supplementary material, further inquiries can be directed to the corresponding author/s.
