# Correction: Roles of SPI-2 T3SS effectors in virulence of *Salmonella* Choleraesuis and construction of a triple-gene mutant vaccine strain

**DOI:** 10.3389/fvets.2026.1840635

**Published:** 2026-04-16

**Authors:** Frontiers Production Office

**Affiliations:** Frontiers Media SA, Lausanne, Switzerland

**Keywords:** *S. choleraesuis*, SPI-2, type III secretion system, virulence, vaccines

In the published article, references 6, 7, 11, 16, 24, 33, 36, 38–41 were assigned incorrect DOIs, while the DOIs for references 13, 21, 22, 23, 30, 32, 34, 37 were not linked to their relevant published articles.

These references have been corrected as follows:

6. Luk-In S, Chatsuwan T, Pulsrikarn C, Bangtrakulnonth A, Rirerm U, Kulwichit W. High prevalence of ceftriaxone resistance among invasive *Salmonella enterica* serotype choleraesuis isolates in Thailand: the emergence and increase of CTX-M-55 in ciprofloxacin-resistant *S*. Choleraesuis isolates. *Int J Med Microbiol*. (2018) 308:447–53. doi: 10.1016/j.ijmm.2018.03.008

7. Jacqueline C, Samper-Cativiela C, Monzon Fernandez S, Ugarte-Ruiz M, Cuesta de la Plaza I, Alvarez J, et al. Phenotypic and genetic characterization of antimicrobial resistance in *Salmonella enterica* serovar Choleraesuis isolates from humans and animals in Spain from 2006 to 2021. *J Antimicrob Chemother*. (2024) 79:790–800. doi: 10.1093/jac/dkae029

11. Ji Z, Shang J, Li Y, Wang S, Shi H. Live attenuated *Salmonella enterica* serovar Choleraesuis vaccine vector displaying regulated delayed attenuation and regulated delayed antigen synthesis to confer protection against *Streptococcus suis* in mice. *Vaccine* (2015) 33:4858–67. doi: 10.1016/j.vaccine.2015.07.063

13. Miao EA, Warren SE. Innate immune detection of bacterial virulence factors via the NLRC4 inflammasome. *J Clin Immunol*. (2010) 30:502–6. doi: 10.1007/s10875-010-9386-5

16. Miao EA, Brittnacher M, Haraga A, Jeng RL, Welch MD, Miller SI. *Salmonella* effectors translocated across the vacuolar membrane interact with the actin cytoskeleton. *Mol Microbiol*. (2003) 48:401–5. doi: 10.1046/j.1365-2958.2003.t01-1-03456.x

21. Edwards RA, Keller LH, Schifferli DM. Improved allelic exchange vectors and their use to analyze 987P fimbria gene expression. *Gene* (1998) 207:149–57. doi: 10.1016/s0378-1119(97)00619-7

22. Zhao X, Dai Q, Zhu D, Liu M, Chen S, Sun K, et al. Recombinant attenuated *Salmonella* Typhimurium with heterologous expression of the *Salmonella* Choleraesuis O-polysaccharide: high immunogenicity and protection. *Sci Rep*. (2017) 7:7127. doi: 10.1038/s41598-017-07689-5

23. Zhao X, Yang F, Shen H, Liao Y, Zhu D, Wang M, et al. Immunogenicity and protection of a pasteurella multocida strain with a truncated lipopolysaccharide outer core in ducks. *Vet Res*. (2022) 53:17. doi: 10.1186/s13567-022-01035-y

24. Zhao X, Zeng X, Dai Q, Hou Y, Zhu D, Wang M, et al. Immunogenicity and protection efficacy of a *Salmonella enterica* serovar Typhimurium fnr, arcA and fliC mutant. *Vaccine* (2021) 39:588–95. doi: 10.1016/j.vaccine.2020.12.002

30. Mandal K. Review of PIP2 in cellular signaling, functions and diseases. *Int J Mol Sci*. (2020) 21:8342. doi: 10.3390/ijms21218342

32. Wang Y, Liu G, Zhang J, Gu D, Hu M, Zhang Y, et al. WbaP is required for swarm motility and intramacrophage multiplication of *Salmonella enteritidis* spiC mutant by glucose use ability. *Microbiol Res*. (2021) 245:126686. doi: 10.1016/j.micres.2020.126686

33. Dumont A, Boucrot E, Drevensek S, Daire V, Gorvel J-P, Poüs C, et al. SKIP, the host target of the *Salmonella* virulence factor SifA, promotes kinesin-1-dependent vacuolar membrane exchanges. *Traffic* (2010) 11:899–911. doi: 10.1111/j.1600-0854.2010.01069.x

34. Feng Z-Z, Jiang A-J, Mao A-W, Feng Y, Wang W, Li J, et al. The *Salmonella* effectors SseF and SseG inhibit Rab1A-mediated autophagy to facilitate intracellular bacterial survival and replication. *J Biol Chem*. (2018) 293:9662–73. doi: 10.1074/jbc.M117.811737

36. Bayer-Santos E, Durkin CH, Rigano LA, Kupz A, Alix E, Cerny O, et al. The *Salmonella* effector SteD mediates MARCH8-dependent ubiquitination of MHC II molecules and inhibits T cell activation. *Cell Host Microbe*. (2016) 20:584–595. doi: 10.1016/j.chom.2016.10.007

37. Dai Y, Zhang M, Liu X, Sun T, Qi W, Ding W, et al. *Salmonella* manipulates macrophage migration via SteC-mediated myosin light chain activation to penetrate the gut-vascular barrier. *EMBO J*. (2024) 43:1499–1518. doi: 10.1038/s44318-024-00076-7

38. Domingues L, Holden DW, Mota LJ. The *Salmonella* effector SteA contributes to the control of membrane dynamics of *Salmonella*-containing vacuoles. *Infect Immun*. (2014) 82:2923–2934. doi: 10.1128/IAI.01385-13

39. Geng S, Wang Y, Xue Y, Wang H, Cai Y, Zhang J, et al. The SseL protein inhibits the intracellular NF-κB pathway to enhance the virulence of *Salmonella pullorum* in a chicken model. *Microb Pathog*. (2019) 129:1–6. doi: 10.1016/j.micpath.2019.01.035

40. Galen JE, Curtiss R. The delicate balance in genetically engineering live vaccines. *Vaccine* (2014) 32:4376–4385. doi: 10.1016/j.vaccine.2013.12.026

41. Pabst O, Cerovic V, Hornef M. Secretory IgA in the coordination of establishment and maintenance of the microbiota. *Trends Immunol*. (2016) 37:287–96. doi: 10.1016/j.it.2016.03.002

The original article has been updated.

