# Supplementary material for: Roles of SPI-2 T3SS effectors in virulence of Salmonella Choleraesuis and Construction of a triple-gene mutant vaccine strain
Source: Front Vet Sci. 2025 Aug 12;12:1637327. doi: 10.3389/fvets.2025.1637327 (PMC12381772; doi:10.3389/fvets.2025.1637327)
Supplement: Supplementary file 1 [file Table_1.docx]

Supplemental Information

Roles of SPI-2 T3SS Effectors in virulence of *Salmonella* Choleraesuis and Construction of a triple-gene mutant vaccine strain

Table S1. Bacterial strains used in this study

| Strains | Description | Source or reference |
| --- | --- | --- |
| **Strains** |  |  |
| CVCC2139  SM10 λ pir | Wild-type *S.* Choleraesuis  *E. coli thi thr-1 leu6 proA2 his-4 arg E2 lacY1 galK2, ara14xyl5 supE44,λpir* | CVCC  ^19^ |
| Δ*sseJ* | Deletion of the *sseJ* in the CVCC2139 | This study |
| Δ*sseG* | *sseG* deletion mutant | This study |
| Δ*sseI* | *sseI* deletion mutant | This study |
| Δ*pipB* | *pipB* deletion mutant | This study |
| Δ*sseL* | *sseL* deletion mutant | This study |
| Δ*slrP* | *slrP* deletion mutant | This study |
| Δ*sopD* | *sopD* deletion mutant | This study |
| Δ*sseK* | *sseK* deletion mutant | This study |
| Δ*spiC* | *spiC* deletion mutant | This study |
| Δ*sopD_2_* | *sopD_2_* deletion mutant | This study |
| Δ*gtgA* | *gtgA* deletion mutant | This study |
| Δ*gtgE* | *gtgE* deletion mutant | This study |
| Δ*ssaV* | *ssaV* deletion mutant | This study |
| Δ*sseF* | *sseF* deletion mutant | This study |
| Δ*sifA* | *sifA* deletion mutant | This study |
| Δ*sifB* | *sifB* deletion mutant | This study |
| Δ*steA* | *steA* deletion mutant | This study |
| Δ*gogB* | *gogB* deletion mutant | This study |
| Δ*steC* | *steC* deletion mutant | This study |
| Δ*pipB_2_* | *pipB_2_* deletion mutant | This study |
| Δ*steD* | *steD* deletion mutant | This study |
| C-Δ*gtgA* | Transform of the pCZb1-Δ*gtgA* into Δ*gtgA* | This study |
| C-Δ*steA* | Transform of the pCZb1-Δ*steA* into Δ*steA* | This study |
| C-Δ*spiC* | Transform of the pCZb1-Δ*spiC* into Δ*spiC* | This study |
| C-Δ*sopD_2_* | Transform of the pCZb1-Δ*sopD_2_* into Δ*sopD_2_* | This study |
| C-Δ*slrP* | Transform of the pCZb1-Δ*slrP* into Δ*slrP* | This study |
| C-Δ*pipB_2_* | Transform of the pCZb1-Δ*pipB_2_* into Δ*pipB_2_* | This study |
| C-Δ*sopD* | Transform of the pCZb1-Δ*sopD* into Δ*sopD* | This study |
| C-Δ*sseF* | Transform of the pCZb1-Δ*sseF* into Δ*sseF* | This study |
| C-Δ*sseJ* | Transform of the pCZb1-Δ*sseJ* into Δ*sseJ* | This study |
| C-Δ*ssaV* | Transform of the pCZb1-Δ*ssaV* into Δ*ssaV* | This study |
| C-Δ*sifB* | Transform of the pCZb1-Δ*sifB* into Δ*sifB* | This study |
| C-Δ*sseK* | Transform of the pCZb1-Δ*sseK* into Δ*sseK* | This study |
| C-Δ*sspH* | Transform of the pCZb1-Δ*sspH* into Δ*sspH* | This study |
| C-Δ*steD* | Transform of the pCZb1-Δ*steD* into Δ*steD* | This study |
| C-Δ*steC* | Transform of the pCZb1-Δ*steC* into Δ*steC* | This study |
| C-Δ*sifA* | Transform of the pCZb1-Δ*sifA* into Δ*sifA* | This study |

Table S2. Plasmids used in this study

| Plasmids | Description | Source or reference |
| --- | --- | --- |
| **Plasmids** |  |  |
| pRE112 | *sacB* mobRP4 R6K ori Cm^+^ | ^21^ |
| pCZb1  pRE112-Δ*sseJ*  pRE112-Δ*sseG*  pRE112-Δ*sseI*  pRE112-Δ*pipB*  pRE112-Δ*sseL*  pRE112-Δ*slrP*  pRE112-Δ*sopD*  pRE112-Δ*sseK*  pRE112-Δ*spiC*  pRE112-Δ*sopD_2_*  pRE112-Δ*gtgA*  pRE112-Δ*gtgE*  pRE112-Δ*ssaV*  pRE112-Δ*sseF*  pRE112-Δ*sifB*  pRE112-Δ*steA*  pRE112-Δ*gogB*  pRE112-Δ*steC*  pRE112-Δ*pipB_2_*  pRE112-Δ*steD*  pRE112-Δ*sspH* | Asd+ Ptrc pSC101 origin, AmpR  For deletion of the *sseJ* gene  For deletion of the *sseG* gene  For deletion of the *sseI* gene  For deletion of the *pipB* gene  For deletion of the *sseL* gene  For deletion of the Δ*slrP* gene  For deletion of the *sopD* gene  For deletion of the *sseK* gene  For deletion of the *spiC* gene  For deletion of the *sopD_2_* gene  For deletion of the *gtgA* gene  For deletion of the *gtgE* gene  For deletion of the *ssaV* gene  For deletion of the *sseF* gene  For deletion of the *sifB* gene  For deletion of the *steA* gene  For deletion of the *gogB* gene  For deletion of the *steC* gene  For deletion of the *pipB_2_* gene  For deletion of the *steD* gene  For deletion of theΔ*sspH* gene | ^20^  This study  This study  This study  This study  This study  This study  This study  This study  This study  This study  This study  This study  This study  This study  This study  This study  This study  This study  This study  This study  This study |
| pCZb1-Δ*gtgA* | Insertion of the *gtgA* into pCZb1 | This study |
| pCZb1-Δ*steA* | Insertion of the *steA* into pCZb1 | This study |
| pCZb1-Δ*spiC* | Insertion of the *spiC* into pCZb1 | This study |
| pCZb1-Δ*sopD_2_* | Insertion of the *sopD_2_* into pCZb1 | This study |
| pCZb1-Δ*slrP* | Insertion of the *sspH* into pCZb1 | This study |
| pCZb1-Δ*pipB_2_* | Insertion of the *pipB_2_* into pCZb1 | This study |
| pCZb1-Δ*sopD* | Insertion of the *sopD* into pCZb1 | This study |
| pCZb1-Δ*sseF* | Insertion of the *sseF* into pCZb1 | This study |
| pCZb1-Δ*sseJ* | Insertion of the *sseJ* into pCZb1 | This study |
| pCZb1-Δ*ssaV* | Insertion of the *ssaV* into pCZb1 | This study |
| pCZb1-Δ*sifB* | Insertion of the *sifB* into pCZb1 | This study |
| pCZb1-Δ*sseK* | Insertion of the *sseK* into pCZb1 | This study |
| pCZb1-Δ*sspH* | Insertion of the *sspH* into pCZb1 | This study |
| pCZb1-Δ*steD* | Insertion of the *steD* into pCZb1 | This study |
| pCZb1-Δ*steC* | Insertion of the *steC* into pCZb1 | This study |
| pCZb1-Δ*sifA* | Insertion of the *sifA* into pCZb1 | This study |

Table S3. Primers used in this study

| Primer name | Forward oligonucleotide(5’-3’) | Reverse oligonucleotide(5’-3’) |
| --- | --- | --- |
| D*sseJ*-F1/R1 | gtcggatgataagtttgtctg | gcagccgatggaactagtgtcctccttactttattaaac |
| D*sseJ*-F2/R2 | agtaaggaggacactagttccatcggctgc | gttcctgctcgccaatg |
| *ssaV*-  F1/R1 | ccaacaacttgatgcgca | agacgttgcatcaatctaaccatgaacgcattgc |
| *ssaV*-  F2/R2 | gcgttcatggttagattgatgcaacgtctgag | ccaggagcagaaaaaatacc |
| *sseG*-  F1/R1 | ggcaggattacccattgc | tatacctgaaaacgatcatggttctccccgag |
| *sseG*-R | cggggagaaccatgatcgttttcaggtatataccgg | gcttttagcggctcatttaaac |
| *sIrp*-  F1/R1 | gggctaagtcagtcagg | aactgcgttcagctaattttccctacctgatctgaac |
| *sIrp*-R | tcaggtagggaaaattagctgaacgcagtttatcag | gctggatgaactggcg |
| *sseF*-  F1/R1 | cacatgaaaataacaatcaataggtatg | gctaacaggtttcatatttcgttctgttatttaagcaataag |
| *sseF*-R | ataacagaacgaaatatgaaacctgttagcccaaatg | cagggagacggctttag |
| *gtgE*-  F1/R1 | gttaataagtggagctttatcccac | ttcggcgagtatatttagttaacctttaccaagataattattcagg |
| *gtgE*-R | ggtaaaggttaactaaatatactcgccgaatagtaattttg | gcagggaaaatcgataacaaac |
| *gogB*-  F1/R1 | gcgcatgaaaataggattcc | atcacacgttaaatatttcattaatggcaagaaaggg |
| *gogB*-R | ttgccattaatgaaatatttaacgtgtgattcatagaaatcg | ctggaggcggattttgtattatc |
| *gtgA*-  F1/R1 | caggagtcttgtcatgcat | cgtttctggaggggagatgagttacacatcctttttactac |
| *gtgA*-R | gatgtgtaactcatctcccctccagaaacgc | cagtatggtgatctcgctg |
| *sifA*-  F1/R1 | gaaagcggtagcgataaagc | ataagtgagattaataatcagacgacgctttctc |
| *sifA*-R | tgattattaatctcacttatactggagtaaaaatg | cagtaaaatgcaaacgttcatcc |
| *sifB*-  F1/R1 | gccattctgactgcaaaatg | ctatttatggtgtggaatgtagaccacaagtggttatatg |
| *sifB*-R | cttgtggtctacattccacaccataaatagtatggatttac | ccacatcttaaaaatgcccc |
| *sseK*-  F1/R1 | gccataacaaattttgcttttgtattac | ttattgccatttccacatgttttttagcgttgcaatc |
| *sseK*-R | acgctaaaaaacatgtggaaatggcaataatcatgctattg | cagagccgttactccatc |
| *steA*-  F1/R1 | ccagaatgatgccgggaatac | attaaggataacacgcagttaacagagtcacgacgataatac |
| *steA*-R | gactctgttaactgcgtgttatccttaattaacgtgttttatg | ggttagttgtcatggtttttaatcg |
| *steC*-  F1/R1 | cccatgacgagataacaaactc | tacgatggagtattacatttagttccgttcttttctgc |
| *steC*-R | gaacggaactaaatgtaatactccatcgtaaaacatctgtag | gcgatgttatggacatgtaattttaac |
| *sseL*-  F1/R1 | cgctattaccctactcaccag | tctaagtactcaccatttgctctgtttcctggtag |
| *sseL*-R | aggaaacagagcaaatggtgagtacttagagcctatc | gatggcacgtatgccttac |
| *sopD_2_*-  F1/R1 | gcaagtgtatcatccatagc | ctttttaatgactttaataactcccttgattatttaccac |
| *sopD_2_*-R | atcaagggagttattaaagtcattaaaaaggcccc | cctggcaggtctgatg |
| *spiC*-  F1/R1 | ctattatatgctgccgtttctg | ttatttactaccattaaatgggagtttctatcaaattcg |
| *spiC*-R | tagaaactcccatttaatggtagtaaataaacgtttaatc | cttaagcgtgtatatacttacactc |
| *sseI*-  F1/R1 | ctgactggccggaaag | acagacgccctccaccctttataaatttacatatagtatctgtccg |
| *sseI*-R | gtaaatttataaagggtggagggcgtctgtc | gcagcagttcgtgcac |
| *pipB*-  F1/R1 | gccctttacaagagacaagac | atccctttatctcgatttgattccttcttatggaagtg |
| *pipB*-R | Taagaaggaatcaaatcgagataaagggattttataaacaagaag | cggaggatagttcatcgtag |
| *sopD*-  F1/R1 | caccatccagtaacccac | ccggattttaaattgcaatatccggagtaaaatatatcgttc |
| *sopD*-R | ttactccggatattgcaatttaaaatccggctgcc | cccggtgatcctcacc |
| *pipB_2_*-  F1/R1 | gtcattcgatatatgacaatgaaagtg | tctgggagaaaatatccctttttgacgtaaatctgaaataaaag |
| *pipB_2_*-R | ttacgtcaaaaagggatattttctcccagagagagcaac | gcgccatgacttcttttaac |
| *steD-*  F1/R1 | caccgtcacgtataatcgg | catgaagaggtttatatgagcaaacacagcaagtc |
| *steD-R* | gctgtgtttgctcatataaacctcttcatgcacatg | ggttgaacaacaatctccag |
| *sspH-*  F1/R1 | cttcaagctccctccaac | gacagatactatatgtgcaccaggtgaatgagg |
| *sspH-R* | cattcacctggtgcacatatagtatctgtccggcag | ggactggtatgggtgcc |
| *gtgA*-F/R | gaaggagatatacatagtgagattaatatgccaacgggaa | tgcaggtctggacattcaattactaaattcgtaggcgattc |
| *steA*-F/R | gaaggagatatacatagtgagattaatatgaagtcatctc | tgcaggtctggacatttacaggtaagagatagtgacg |
| *spiC*-F/R | gaaggagatatacatagtgagattaatatgtctgaggagg | tgcaggtctggacatttataccccacccgaataaag |
| *sopD_2_*-F/R | gaaggagatatacatagtgagattaatatgccagttacg | tgcaggtctggacatttatataagcatattgcgacaactc |
| *sIrp*-F/R | aaggagatatacatagtgagattaatatgtttaatattactaatatacaatc | tgcaggtctggacattcaccagtaggcgctc |
| *pipB_2_*-F/R | gaaggagatatacatagtgagattaatatgcagcgttc | tgcaggtctggacatctaaatattttcactataaaattcgttaaagag |
| *sopD*-F/R | gaaggagatatacatagtgagattaatatgccagtcac | tgcaggtctggacatttatgtcagtaatatattacgactgcac |
| *sseF*-F/R | gaaggagatatacatagtgagattaatatgaaaattcatattccg | tgcaggtctggacattcatggttctccccgag |
| *sseJ*-F/R | gaaggagatatacatagtgagattaatatgccattgagtg | tgcaggtctggacatttattcagtggaataatgatgagc |
| *ssaV*-F/R | gaaggagatatacatagtgagattaatatgcgttcatgg | tgcaggtctggacattcattcttcattgtccgcc |
| *sifB*-F/R | gaaggagatatacatagtgagattaatatgccaattactatc | tgcaggtctggacattcaactctggtaatgagcctc |
| *steD*-F/R | gaaggagatatacatagtgagattaatatgaatgtcacttcag | tgcaggtctggacatttatggccaggctggc |
| *sspH*-F/R | gaaggagatatacatagtgagattaatatgccctttcatattg | tgcaggtctggacattcagttacgacgccactg |
| *sifA*-F/R | gaaggagatatacatagtgagattaatatgccgattac | tgcaggtctggacatttataaaaaacaacataaacagccg |
| *sseK*-F/R | gaaggagatatacatagtgagattaatatggagcatttaattg | tgcaggtctggacatctactgcacatgcctagc |
| *steC*-F/R | gaaggagatatacatagtgagattaatgtgaccgagac | gcaggtctggacatctatttttttaattcattctttaatactttagcc |
| pCZb1-F/R | atgtccagacctgcagcc | atgtatatctccttcttaaatctagag |
| pRE112-F/R | CACTTATTCAGGCGTAGCAAC | GAGCTGCATGACAAAGTCATC |
